# Metabolomic Profiling, Antioxidant and Enzyme Inhibition Properties and Molecular Docking Analysis of Antarctic Lichens

**DOI:** 10.3390/molecules27228086

**Published:** 2022-11-21

**Authors:** Alfredo Torres-Benítez, José Erick Ortega-Valencia, Marta Sanchez, Pradeep Kumar Divakar, Mario J. Simirgiotis, María Pilar Gómez-Serranillos

**Affiliations:** 1 Instituto de Farmacia, Facultad de Ciencias, Campus Isla Teja, Universidad Austral de Chile, Valdivia 5090000, Chile; 2 Tecnológico Nacional de México/Instituto Tecnológico de Tlalnepantla, Av. Instituto Tecnológico, S/N. Col. La Comunidad, Tlalnepantla de Baz 54070, Mexico; 3 Departamento de Farmacología, Farmacognosia y Botánica, Facultad de Farmacia, Universidad Complutense de Madrid, Plaza Ramón y Cajal s/n, Ciudad Universitaria, 28040 Madrid, Spain

**Keywords:** *Lecania*, *Pseudephebe*, *Sphaerophorus*, bioactive compounds, antioxidant, enzyme inhibition, Antarctica, neuroprotective potential

## Abstract

The lichen species *Lecania brialmontii*, *Pseudephebe pubescens*, and *Sphaerophorus globosus* are part of the prominent lichenoflora of the Antarctic territory. In this work, we report the metabolomic identification of ethanolic extracts of these species, their antioxidant and cholinesterase enzyme inhibitory activity, and conduct a molecular docking analysis with typical compounds. Eighteen compounds were identified by UHPLC-ESI-QTOF-MS in *L. brialmontii*, 18 compounds in *P. pubescens*, and 14 compounds in *S. globosus*. The content of phenolic compounds was variable among the species, ranging from 0.279 to 2.821 mg AG/g, and all three species showed high inhibition potential on the cholinesterase enzymes. Molecular docking showed important interactions between AChE and BChE with the selected compounds. This study evidences the chemical fingerprint of three species of the order Lecanorales that support the continuation of the study of other biological activities and their potential for medical research.

## 1. Introduction

Lichens are a symbiotic association formed by the interaction between a photosynthetic organism (algae or cyanobacteria), an ascomycete or basidiomycete fungus, and the association of a microbiome [1,2], and, due to their ecological strategy of symbiosis, they can be found in a variety of environments, from deserts to rainforests and moors, and even in aquatic ecosystems [3]. These organisms can develop on different substrates, including bark, rock, soil, and leaves, but much of the water and nutrients are taken directly from the atmosphere [4]. Their general physiology, resulting from the characteristics of symbionts, has allowed them to be recognized as indicators of environmental quality, due to their high sensitivity to changes in atmospheric components, which affect their abundance, distribution, and vitality [5].

**Figure 1:**
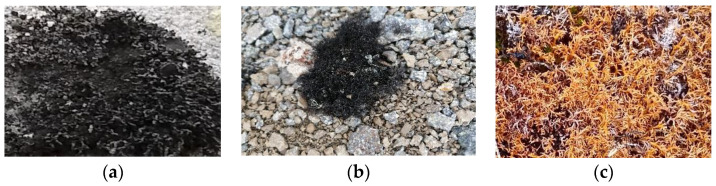
Lichens thallus in the polar tundra of King George Island, Maritime Antarctic (**a**) *Lecania brialmontii*; (**b**) *Pseudephebe pubescens*; (**c**) *Sphaerophorus globosus*.

The Antarctic continent has an area of approximately 13,500,000 km^2^, mostly covered by permanent ice caps. Plant and fungal organisms develop to a limited extent in coastal sectors free of ice and snow during the summer period, and in rocky outcrops in the interior of the territory. Lichens are the group with the greatest specific diversity and adaptation to Antarctic conditions, due to their psychrophilic capacity that allows them to carry out biochemical and metabolic processes [6,7]. As for lichen species of the order Lecanorales, distributed in the archipelago of the South Shetland Islands (Maritime Antarctica), we find *Lecania brialmontii* (Vain.) Zahlbr (family Ramalinaceae), with dwarf-fruticose thallus, 6 to 13 mm high, cylindrical, lateral apothecia, with continuous or crenulate margin, brownish-dark disc, saxicolous. We also find *Pseudephebe pubescens* (L.) M. Choisy (family Parmeliaceae), with fruiting thallus, cylindrical, black, with dichotomous branches, flat at the ends, saxicolous and *Sphaerophorus globosus* (Huds.) Vain. (family Sphaerophoraceae), with fruticulose thallus, forming colonies, sympodial ramifications, on the ground between mosses and lichens. There are limited studies on these species, despite their representing a source of knowledge at the taxonomic, ecological and biological levels of the lichen community present on the white continent (Figure 1) [6,8,9,10].

Recent research in the area of medicinal chemistry has focused its efforts on finding natural compounds from species of multiple lineages and taxa with high biological potential at the level of extracts or bioactive compounds. In lichens, phenolic-type compounds, such as depsides, depsidones, depsones, lactones, dibenzofurans, anthraquinones, pulvinic acid derivatives and xanthones, have been especially identified [11,12,13], evidencing activities of antioxidant, anti-inflammatory, anticancer, antimutagenic, and antifungal types, among others [14,15,16]. The potential of these compounds to counteract oxidative stress was reported in [17,18,19], highlighting the action of atranorin, usnic acid, lecanoric acid, diffractaic acid, lobaric acid, stictic acid, furmarprotocetraric acid, salazinic acid, physodic acid, evernic acid, gyrophoric acid, 3-hydroxyphysodic acid, physodalic acid, vesuvianic acid, sekikaic acid, isidiophorin, rhizonyl alcohol, atranol, chloroatranol, orsellinic acid and other orsellinates, mostly present in lichen species of the families Cladoniaceae, Parmeliaceae, Lobariaceae, Lecanoraceae, Ramalinaceae, Umbilicariacea and others [20]. The antioxidant action is variable, according to the level of solubility of the extract used for its evaluation and isolation of compounds, as well as the presence of components, such as proteins and carbohydrates, and is evaluated through correlation with assays, such as DPPH, ORAC, and the determination of total phenol content [21,22]. Furthermore, the evaluation of inhibitory activity on cholinesterase enzymes (AChE and BChE), as a therapeutic target for neurodegenerative diseases, was reported in species of the genera *Allocetraria*, *Asahinea*, *Cetraria*, *Dactylina*, *Nephromopsis*, *Tuckermannopsis*, *Tuckneraria* and *Vulpicida* [23].

In recent years, for the characterization of secondary metabolites in lichen extracts, the ultra-high-resolution chromatography (UHPLC) technique, coupled to a mass spectrometer, has been used to obtain diagnostic fragments for each type of compound, forming a robust and accurate tool for the chemical study of lichen extracts [24,25,26,27,28]. In this work, we report the metabolomic identification of the ethanolic extracts of *L. brialmontii*, *P. pubescens*, and *S. globosus*, their antioxidant and cholinesterase enzyme inhibitory activity, and conduct a molecular docking analysis with typical compounds in the study species. Virtual structure-based screening (VS) is a widely used process, that uses the properties and knowledge of the three-dimensional structure of the protein of interest with the aim of designing new organic molecules to be used as potential drugs [29]. This process evaluates, through predictions, the possible unions and affinities of the organic molecules within the binding site of the protein of interest, which facilitates an understanding of the important properties related to the binding process. Empirical scoring functions are widely used for posing and affinity prediction [29,30]. Although pose prediction is performed with satisfactory accuracy, correct binding affinity accuracy remains a challenging and crucial task for the success of structure-based VS experiments. However, these scores allow evaluation of the possible behavior of the organic molecule at the protein binding site. We know that for the study of molecular coupling there are various software that can be used, that can have a higher performance in the identification and qualification of molecular coupling [31]. Therefore, in this study, it was decided to perform the molecular docking analyses at UCSF Chimera using Autodock Vina tools.

## 2. Results and Discussion

### 2.1. Metabolomics Profiles and Chemical Fingerprints of Lichen Extracts

#### 2.1.1. UHPLC Chromatographic Analysis of *Lecania brialmontii*

**Figure 2:**
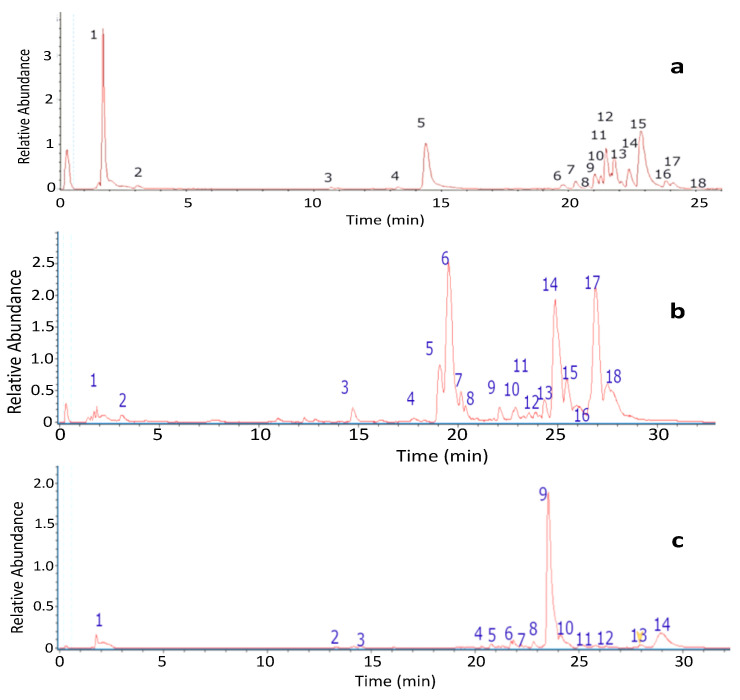
UHPLC-MS Chromatograms (**a**) *Lecania brialmontii*; (**b**) *Pseudephebe pubescens*; (**c**) *Sphaerophorus globosus*.

The fingerprinting of the ethanolic extract of *L. brialmontii* was obtained by means of high-resolution mass spectrometric analysis (UHPLC-MS). The negative mode was used for the tentative identification of the eighteen peaks (Figure 2). The metabolites identified in this species were carbohydrates, acids, anthraquinones, aromatics, lipids and depsides (Table 1).

##### Carbohydrates

Peak 1 was identified as mannitol (C_3_H_13_O_6_).

##### Fatty Acids

Peak 2 was identified as citric acid (C_6_H_7_O_7_).

##### Anthraquinones

Peaks 3 and 7 were identified as swertianin (C_14_H_9_O_6_) and rhein (C_15_H_7_O_6_), respectively.

##### Aromatic Derivatives

Peak 4, with a molecular anion at *m*/*z* 179.0344 and diagnostic peaks at *m*/*z* 107.0488, 135.0437, and 151.0386, was identified as 2,4-diformyl-3,5-dihydroxytoluene or 2,6-diformyl-3,5-dihydroxytoluene (C_9_H_7_O_4_), while peak 5 was identified as 5,7-dihydroxy-6-methylphtalide (C_9_H_7_O_4_) and peak 6 was identified as atranol (C_8_H_7_O_3_), with a molecular anion at *m*/*z* 151.0395 and diagnostic peaks at *m*/*z* 135.0438, 123.0438, and 107.0488. These compounds were also reported in Antarctic lichens, such as *Himantormia lugubris* by Areche et al. [28], and in species of the genera *Parmotrema, Sticta* and *Usnea* [24,25,26].

##### Lipids

Peak 8 was identified as 9,10,12,13-tetrahydroxyheneicosanoic acid (C_21_H_41_O_6_); peak 9 as tetrahydroxypentacosanedioic acid (C_25_H_47_O_8_); peak 10 as 9,10,12,13-tetrahydroxydocosanoic acid (C_22_H_43_O_6_); and peak 11 as pentahydroxyoxohexacosanoic acid (C_26_H_49_O_8_). Peak 14 was identified as 9,10,12,13-tetrahidroxytricosanoic acid (C_23_H_45_O_6_) and peak 15 as 9,10,12,13,14,15-hexahydroxyheptacosenoic (C_27_H_51_O_8_), with diagnostic peaks at *m*/*z* 475.3615, 443.3355, and 371.0377, while peak 16 was identified, with an [M−H]^−^ ion at *m*/*z* 517.3740, as the related methyl-9,10,11,12,13-pentahydroxy-14-oxoheptacosanoate (C_28_H_53_O_8_). These compounds agreed with a previous report by Areche et al. [28].

##### Depsides

Peak 12, with a molecular anion at *m*/*z* 331.0818 and diagnostic peaks at *m*/*z* 135.0438, 123.0439, 181.0494, 151.0386, 167.0336, and 313.0703, was identified as evernic acid (C_17_H_15_O_7_). Peak 13 was identified as brialmontin 2 (C_24_H_47_O_7_) and peak 17, with a molecular anion at *m*/*z* 317.0666, was identified as lecanoric acid (C_16_H_13_O_7_); while peak 18was identified as barbatic acid (C_19_H_19_O_7_), with diagnostic peaks at *m*/*z* 181.0493, 163.0387, and 137.0594. Depsides are particular compounds in lichens. Evernic acid was reported to have neuroprotective effects, due to its participation in the regulation of neuronal mitochondrial function and neuroinflammation, in [32]. Bryalmontin 2, as a poly-substituted despside, was reported to be a compound unique to the genus *Lecania* [33]. The compounds lecanoric acid and barbatic acid are widely reported in tropical, austral, and Antarctic lichens [24,25,26,28].

The genus *Lecania* has been a focus of study to objectively define its species through molecular phylogeny studies using nucleotide sequences from the mt-SSU rRNA, the ITS region of the nu-rDNA, and the RNA polymerase II second largest subunit [34]. However, the characterization of the chemical fingerprint of the species could contribute to elucidate their similarities and differences and new groupings. On the other hand, *L. brialmontii*, together with species of the genera *Usnea, Polycauliona* and *Cladonia*, have been reported as a source of leavenings that produce extracellular hydrolytic enzymes and are of interest in biotechnological applications of catalytic origin [35].

#### 2.1.2. UHPLC Chromatographic Analysis of *Pseudephebe pubescens*

The fingerprinting of the ethanolic extract of *P. pubescens* was obtained by means of high-resolution mass spectrometric analysis (UHPLC-MS). The negative mode was used for the tentative identification of the eighteen peaks (Figure 2). The metabolites identified in this species were carbohydrates, acids, lipids, depsides, aromatics and dibenzofurans (Table 2).

##### Carbohydrates

Peak 1 was identified as mannitol (C_6_H_13_O_6_). Peak 4, with a molecular anion at *m*/*z* 245.0484, was identified as visnagin (C_13_H_9_O_5_); while peak 5 was identified, with an [M−H]^−^ ion at *m*/*z* 245.0489, as the related khellinol (C_13_H_9_O_5_).

##### Fatty Acids

Peak 2 was identified as citric acid (C_6_H_7_O_7_).

##### Lipids

Peak 3 was identified as azelaic acid (C_9_H_15_O_4_); while peak 6 was identified as 9-octadecenedioic acid (C_18_H_31_O_4_) and peak 7 was tentatively identified as pinellic acid (C_18_H_33_O_5_). Peak 8 was identified as olivetolic acid (C_12_H_15_O_4_) and peak 9 as pentahydroxyoxohexacosanoic acid (C_26_H_49_O_8_), with diagnostic peaks at *m*/*z* 403.3001, and 979.6848. Peak 14 was identified as 17-hydroxylinolenic acid (C_18_H_29_O_3_) and peak 15 as porrigenic acid (C_18_H_29_O_4_). Peaks 17 and 18 were identified as 18-hydroxylinoleic acid (C_18_H_31_O_3_) and 18-hydroxylinolenic acid (C_18_H_29_O_3_), respectively. These compounds were previously reported in tropicales, austral and Antarctic lichen species [24,25,27].

##### Depsides

Peak 10 was identified as lecanoric acid (C_16_H_13_O_7_) and peak 11 was identified as tetrahydroxytricosanoic acid (C_23_H_46_O_6_) [24,25,26,28]. 

##### Aromatic Derivatives

Peak 12 was identified as 3,5-diethoxybenzoic acid (C_11_H_13_O_4_) and peak 13, with a molecular anion at *m*/*z* 417.1553, as sekikaic acid (C_22_H_25_O_8_) [24,25].

##### Dibenzofurans

Peak 16 was identified as usnic acid (C_18_H_15_O_7_), with diagnostic peaks at *m*/*z* 295.2291, 231.0647, and 328.0570. This compound was reported in previous studies and is recognized for multiple biological activities in in vitro and in vivo assays [36].

The genus *Pseudephebe* has been of great interest for studies of species delimitation and biogeographic processes, based on molecular tools and microevolutionary analyses that facilitate the defining of the origin of its distribution [37]. Therefore, these species represent a model for the study of biological, ecological, chemical, and systematic variables.

#### 2.1.3. UHPLC Chromatographic Analysis of *Sphaerophorus globosus*

The fingerprinting of the ethanolic extract of *S. globosus* was obtained by means of high-resolution mass spectrometric analysis (UHPLC-MS). The negative mode was used for the tentative identification of the fourteen peaks (Figure 2). The metabolites identified in this species were carbohydrates, aromatics, acids, depsides and dibenzofurans (Table 3).

##### Carbohydrates

Peak 1 was identified as mannitol (C_6_H_13_O_6_).

##### Aromatic Derivatives

Peak 2 was identified as vanillic acid (C_8_H_8_O_4_) and peak 3 as vanilloloside (C_14_H_19_O_8_). Peak 4, with a molecular anion at *m*/*z* 181.0501 and diagnostic peaks at *m*/*z* 151.0387, 123,0439, and 135.0438, was identified as methyl orsellinate (C_9_H_9_O_4_); while peak 5 was identified as 2,6-diformyl-3,5-dihydroxytoluene (C_9_H_7_O_4_), with a molecular anion at *m*/*z* 179.0344 and diagnostic peaks at *m*/*z* 151.0386, 107.0488, and 135.0437. Peak 9 was identified as 6-heptylresorcylic acid (C_14_H_19_O_4_) and peak 13 as acetoxyisovalerylalkannin (C_23_H_25_O_8_). Peak 14 was identified as 2′-*O*-methyldivaricatic acid (C_22_H_25_O_7_) [24,25].

##### Fatty Acids

Peak 6 was identified as protolichesterinic acid (C_19_H_31_O_4_).

##### Depsides

Peak 7, with a molecular anion at *m*/*z* 431.1657 and diagnostic peaks at *m*/*z* 417.15290, 401.08231, and 267.1228, was identified as 4′-*O*-methyl norhomosekikaic acid (C_23_H_27_O_8_); peak 8, with daughter ions at *m*/*z* 233.1166; 207.1376 and 251.1275, was identified as sphaerophorin (C_23_H_27_O_7_); peak 10, with an [M−H]^−^ ion at *m*/*z* 317.0666, was identified as lecanorid acid (C_16_H_13_O_7_); while peak 11 with a molecular anion at *m*/*z* 417.1554 1657 and diagnostic peaks at *m*/*z* 267.1228, 251.1289, and 285.09033, was identified as sekikaic acid (C_22_H_25_O_8_) [32,33,34,36]. The compound sphaerophorin is reported to have a protective effect against human melanoma cells, inhibiting their growth and promoting apoptotic cell death [38].

##### Dibenzofurans

Peak 12 was identified as usnic acid (C_18_H_15_O_7_), with diagnostic peaks at *m*/*z* 295.2291, 231.0647, and 328.0570. This compound is widely reported in metabolomics studies on lichens [24,25,28].

In general, previous studies of the chemical composition of *S. globosus* extracts have evidenced chemopreventive potential in cancer, such as blocking estrogen formation by inhibition of the aromatase enzyme [39]. Likewise, the intervention of this lichen in primary colonization processes [40], exhibits a potential for further study, to correlate the presence of compounds with ecological advantages.

### 2.2. Total Phenolic Contents and Antioxidant Activity

The range of polyphenolic content present in ethanol extract in the three lichen species ranged from 0.279 to 2.821 mg AG/g and showed moderate antioxidant activity (Table 4). The species *P. pubescens* showed a higher presence of phenolic compounds. On the other hand, *S. globosus* presented a higher oxygen radical absorption capacity, compared to the higher reducing power of *P. pubescens*.

The total phenolic content was relatively low. However, these results coincided with previous reports where species such as *P. pubescens* showed low content of phenolic compounds, but high concentrations of polysaccharides and proteins [41]. The antioxidant activity of the ethanolic extracts was high, and in species such as *P. pubescens* and *S. globosus*, high values of superoxide anion trapping were previously reported with methanolic and acetone extracts [42]. These results are in addition to the significant antioxidant capacity reported in extracts and compounds of lichen species of the genera *Toninia*, *Usnea*, *Parmelia*, *Flavoparmelia*, *Evernia*, *Hypogymnia*, *Cladonia*, *Vulpicida*, *Pseudevernia* and *Cetraria* [19,43,44,45].

### 2.3. Enzymatic Inhibitory Activity

The values obtained with the ethanolic extracts of the three lichen species (Table 5), showed high inhibition potential on the cholinesterase enzymes. For AChE, the inhibition ranged from 3949 to 10,422 µg/mL, while for BChE it ranged from 4476 to 8828 µg/mL.

These results are comparable with the work reported with the ethanolic extract of the Antarctic species *Himantormia lugubris* in [28], which showed an inhibitory activity on AChE of 12.38 ± 0.09 µg/mL and on BChE of 31.54 ± 0.20 µg/mL, while the ethanolic extracts of *L. brialmontii*, *P. pubescens* and *S. globosus* showed a higher potential for inhibition of cholinesterase enzymes. These data reported better inhibition efficacy against *Cladonia uncinalis* extract, which weakly inhibited AChE, and, for BChE, the extracts of *Parmelia sulcata* and *C. uncinalis* showed inhibition values of 42.9 ± 0.1 µg/mL and 85.9 ± 0.2 µg/mL, respectively [46]. On the other hand, the inhibiting activity in the endogenous fungus *Diaporthe mehothocarpus* from the lichen *Cladonia symphycarpia* [47] was much lower compared to the results obtained in the three study species.

### 2.4. Docking Studies

The compounds present and typical in the extracts of *Lecania brialmontii*, *Pseudephebe pubescens* and *Sphaerophorus globosus*, as well as the known inhibitor of acetylcholinesterase and butyrylcholinesterase, were used for molecular docking assays in order to analyze the protein molecular interactions with the main amino acid residues involved in the inhibition of these structures. The best coupling binding affinities for each of the ligands were expressed in kcal/mol and compared with the coupling energy of the inhibitor galantamine (Table 6).

#### 2.4.1. Acetylcholinesterase (TcAChE) Docking Results

**Figure 3:**
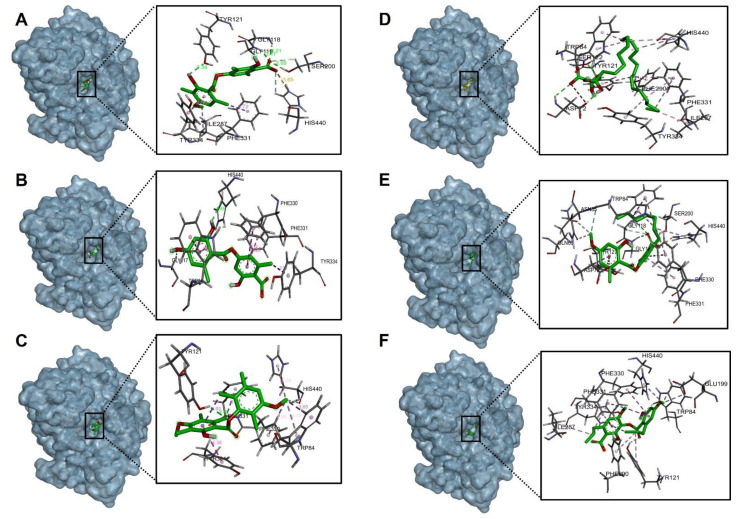
Binding mode and predicted intermolecular interactions of selected compounds from *Lecania brialmontii*, *Pseudephebe pubescens* and *Sphaerophorus globosus* extracts and residues of the Torpedo californica acetylcholinesterase (TcAChE) catalytic site; (**A**) Barbatic acid at the catalytic site; (**B**) Lecanoric acid at the catalytic site; (**C**) Brialmontin 2 at the catalytic site; (**D**) Tetra-hydroxytricosanoic acid at the catalytic site; (**E**) Sphaerophorin at the catalytic site; (**F**) Sekikaic acid at the catalytic site.

**Figure 4:**
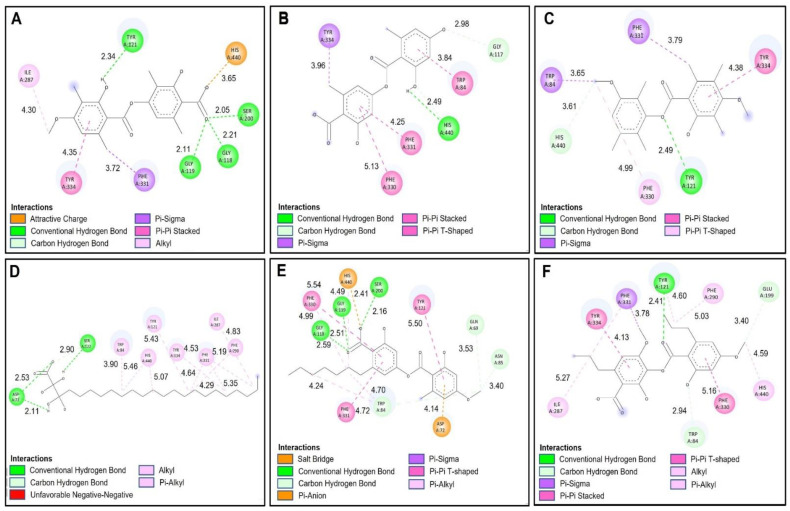
Two-dimensional diagram of compounds from *Lecania brialmontii*, *Pseudephebe pubescens* and *Sphaerophorus globosus* extracts and residues of the Torpedo californica acetylcholinesterase (TcAChE) catalytic site; (**A**) Barbatic acid at the catalytic site; (**B**) Lecanoric acid at the catalytic site; (**C**) Brialmontin 2 at the catalytic site; (**D**) Tetrahydroxytricosanoic acid at the catalytic site; (**E**) Sphaerophorin at the catalytic site; (**F**) Sekikaic acid at the catalytic site. Yellow dotted lines indicate hydrogen bond interactions; cyan dotted lines represent π-π interactions; magenta dotted lines represent T-shape; and red dotted lines indicate salt bridge interactions.

Table 6 shows the binding affinities of the compounds barbatic acid, lecanoric acid, brialmontin 2, tetrahydroxytricosanoic acid, sphaerophorin and sekikaic acid obtained from the extracts of *Lecania brialmontii*, *Pseudephebe pubescens* and *Sphaerophorus globosus*. Most of these compounds presented good energy descriptors on the enzyme acetylcholinesterase. Barbatic acid and sphaerophorin were arranged in the same way at the catalytic site of acetylcholinesterase (Figure 3A,E), which could probably explain the similarity in their binding affinities (−10.30 kcal/mol and −9.50 kcal/mol, respectively). Likewise, both compounds presented the same hydrogen bonds between the carbonyl group of the carboxylate in their structures and the residues Ser200, Gly118 and Gly119 (Figure 4A,E). Similarly, it was observed that the two compounds interacted with the main residues in the catalytic site, which were Ser200 and His400; thus, allowing greater stability in the catalytic site of acetylcholinesterase. However, barbatic acid presented a binding affinity very similar to that of the reference inhibitor galantamine. In addition to the interactions of hydrogen bonds that the barbatic acid presented, it showed π-π and π-sigma type interactions with the Tyr334 and Phe331 residues, respectively (Figure 4A).

Tetrahydroxytricosanoic acid had the lowest binding affinity (Table 6); this was due to the number of rotatable bonds that its molecular structure presented, causing its molecular geometry to be less stable in the catalytic site of acetylcholinesterase. It was observed that the tetrahydroxytricosanoic acid presented 2 interactions of hydrogen bonds between the oxygen atom of the carboxylate and the Ser122 residue, and the alpha carbon hydroxyl group with the Asp72 residue (Figure 3D and Figure 4D). However, the interactions that the aliphatic chain of tetrahydroxytricosanoic acid presented were very weak, which meant that it did not have good disposition and stability in the binding site with acetylcholinesterase.

Brialmontin 2 and sekikaic acid were arranged in a very similar way in the catalytic site of acetylcholinesterase (Figure 3C,F), and very similar binding energies (−9.80 kcal/mol and −9.30 kcal/mol) and very similar interactions with some amino acid residues was observed. However, these two compounds presented some differences. The two compounds presented hydrogen bond type interactions with the Tyr121 residue. However, brialmontin 2 did so with the oxygen atom of the ester group of its structure, while sekikaic acid performed the interaction with the oxygen atom of the group ester carbonyl (Figure 4C,F). These two compounds presented π-π T-shaped interactions with the Phe330 amino acid, but with the difference that in brialmontin 2 it was present between the methoxyl group of the aromatic ring and the aromatic ring of the Phe330 residue, while with sekikaic acid it was carried out between the ring aromatic part of its structure and the aromatic ring of the Phe330 residue.

Lecanoric acid was the second compound that presented a higher binding affinity for acetylcholinesterase because it presented hydrogen bond type interactions between the hydroxyl group of the aromatic ring and the His400 residue (Figure 3B and Figure 4B), which is one of the important residues in the catalytic site of acetylcholinesterase, giving it high affinity and, therefore, high binding affinity.

#### 2.4.2. Butyrylcholinesterase (hBChE) Docking Results

**Figure 5:**
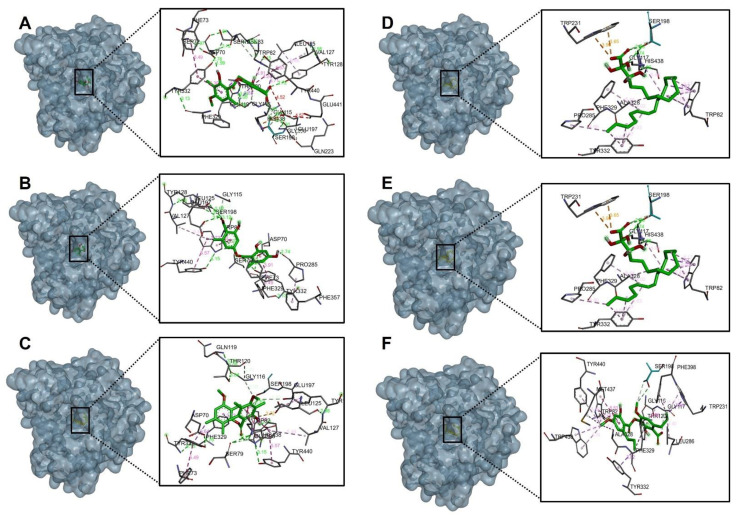
Binding mode and predicted intermolecular interactions of selected compounds from *Lecania brialmontii*, *Pseudephebe pubescens* and *Sphaerophorus globosus* extracts and residues of the human butyrylcholinesterase (hBChE) catalytic site; (**A**) Barbatic acid at the catalytic site; (**B**) Lecanoric acid at the catalytic site; (**C**) Brialmontin 2 at the catalytic site; (**D**) Tetrahydroxy-tricosanoic acid at the catalytic site; (**E**) Sphaerophorin at the catalytic site; (**F**) Sekikaic acid at the catalytic site.

**Figure 6:**
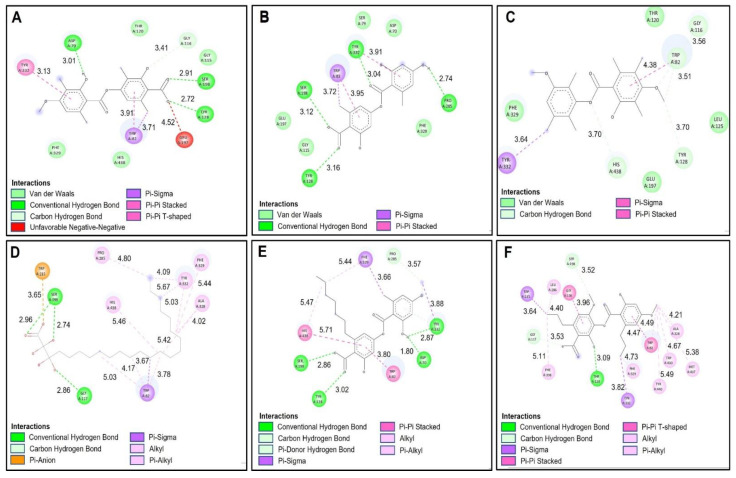
Two-dimensional diagram of compounds from *Lecania brialmontii, Pseudephebe pubescens* and *Sphaerophorus globosus* extracts and residues of the human butyrylcholinesterase (hBChE) catalytic site; (**A**) Barbatic acid at the catalytic site; (**B**) Lecanoric acid at the catalytic site; (**C**) Brialmontin 2 at the catalytic site; (**D**) Tetrahydroxytricosanoic acid at the catalytic site; (**E**) Sphaerophorin at the catalytic site; (**F**) Sekikaic acid at the catalytic site. Yellow dotted lines indicate hydrogen bond interactions; cyan dotted lines represent π-π interactions; magenta dotted lines represent T-shape; and red dotted lines indicate salt bridge interactions.

The molecular isolation results between the selected compounds of the extract of *Lecania brialmontii*, *Pseudephebe pubesecens* and *Sphaerophorus globosus* and butyrylcholinesterase (hBChE) showed lower binding affinities, compared to the results obtained with acetylcholinesterase (Table 6). It was observed that most of the compounds exhibited slightly higher binding affinities compared to the reference inhibitor galantamine. However, tetrahydroxytricosanoic acid stood out as the compound with the lowest binding affinity compared to the other compounds (Table 6) and this behavior was observed in the same way in the results obtained with acetylcholinesterase (TcAChE). As mentioned above, this exchange was highly influenced by the stability of the structure, since having a high number of rotatable bonds prevented it from having a better conformation in the catalytic site of butyrylcholinesterase (hBChE) (Figure 5D). However, it was observed that tetrahydroxytricosanoic acid showed hydrogen bonds with residues Ser198 and Gly117 (Figure 6D). Although these residues were involved in the inhibition of butyrylcholinesterase (hBChE), the instability of tetrahydroxytricosanoic acid caused presentation of a lower binding affinity compared to the other compounds.

Lecanoric acid showed better binding affinity results with butyrylcholinesterase (hBChE), due to its interactions with the main residues of the catalytic site responsible for inhibition of butyrylcholinesterase (hBChE). Figure 6B shows that lecanoric acid exhibited 4 hydrogen bonds, one of which was between the oxygen atom of the carboxylate and the hydrogen of Ser198. The next hydrogen bond interaction was between the carbonyl group of the carboxylate and the Tyr128 residue, and another hydrogen bond interaction was between the group phenolic ring hydroxide and the Pro285 residue. The last hydrogen bonding interaction was between the ester cabonyl group with the Tyr332 residue. These interactions favored the blocking of lecanoric acid within the catalytic site (Figure 5B), which generated greater stability and, therefore, a better binding affinity with butyrylcholinesterase (hBChE).

Barbatic acid had a lower binding affinity for butyrylcholinesterase (hBChE) than for acetylcholinesterase (TcAChE). This was, mainly because the carboxylate group had a negative interaction with the Glu197 residue (Figure 6A), which caused its stability to decrease at the site catalytic of butyrylcholinesterase (hBChE), so it presented lower binding affinity. However, it was observed that it showed hydrogen bond type interactions with one of the residues responsible for the inhibition of butyrylcholinesterase (hBChE), which was the Ser198 residue (Figure 5A and Figure 6A).

Sphaerophorin and galantamine were shown to have very similar binding affinities (−8.70 kcal/mol and −8.80 kcal/mol, respectively), since sphaerophorin interacted well with the catalytic site of butyrylcholinesterase (hBChE) (Figure 5E and Figure 6E). As in the other compounds, the carboxylate group presented favorable interaction of the hydrogen bond type with the residues Ser198 and Tyr128 (Figure 6E). The phenolic ring hydroxyl group exhibited two hydrogen bonding interactions with residues Tyr332 and Asp70. Interactions of the π-sigma type were observed between the methoxyl group of the aromatic ring and residue Phe329, and, in addition, π-π type interactions between the aromatic ring and residues His438 and Trp82 were observed (Figure 6E). These interactions favored sphaerophorin presenting stability in the catalytic site, confirming good binding affinity (Figure 5E and Table 6).

Brialmontin 2 presented π-sigma interactions between the methyl group of the aromatic ring and residue Tyr332, as well as carbon-hydrogen bond type interactions between the oxygen atom of the ester with residue His438, the oxygen atom and the methyl of the group methoxyl with residue Trp82 (Figure 5C and Figure 6C). Figure 5F and Figure 6F show the interactions that sekikaic acid presented with butyrylcholinesterase (hBChE). Hydrogen bond type interactions were present between the hydroxyl of the aromatic ring with the residue Thr120. Signals of π-sigma type were observed between the methyl groups of the propyl attached to the aromatic rings and the residues Trp231 and Tyr332 and π-π interactions were present between residues Gly116 and Trp82 with the aromatic rings (Figure 5F and Figure 6F).

### 2.5. Prediction of Pharmacokinetic Properties—ADME

The phytochemicals extracted from *Lecania brialmontii*, *Pseudephebe pubesecens* and *Sphaerophorus globosus* were subjected to pharmacokinetic analysis comparing them with the cholinesterase inhibitor galantamine, using the Osiris Data Warrior program (Table 7). According to Lipinski’s ‘rule of five’, a good drug candidate for consideration in preclinical studies should have a molecular weight (MW) ≤ 500 Da, number of gyratory bonds ≤ 10, number of acceptor hydrogen bonds ≤ 10, number of hydrogen bond donors ≤ 5 and a Clog *p* value ≤ 5 [48,49]. As shown in Table 7, the only phytochemical that did not meet the criteria was tetrahydroxytricosanoic acid, due to its higher number of rotatable bonds and having cLogP greater than five. These results were highly related to the docking analysis, since tetrahydroxytricosanoic acid presented lower binding affinities in cholinesterases.

The bioavailability of the phytochemicals was evaluated by TPSA analysis. This parameter was highly related to passive molecular transport through membranes and to assessing the bioavailability of phytochemicals. From the TPSA values, the absorption percentages were calculated using Equation (1) [50]. The best absorption percentage values were presented by Brialmontin 2 (86.59%), because it presented a lower TPSA compared to the other phytochemicals. However, most of the phytochemicals presented absorption percentages greater than 60% (Table 7), indicating good bioavailability.

### 2.6. Toxicity Prediction

The pharmacodynamic (toxicological) properties of the phytochemicals extracted from *Lecania brialmontii, Pseudephebe pubescens* and *Sphaerophorus globosus* were evaluated using the Osiris Data Warrior computational tool. The toxicity risks that were evaluated were mutagenicity, tumorigenicity, irritation and reproductive toxicities (Table 8). The results showed that the only phytochemicals with toxicity risks were Brialmontin 2 and tetrahydroxytricosanoic acid. Brialmontin presented high levels of tumorigenicity and irritation since, when metabolized, one of the aromatic rings that contains two methyl groups, conferred the risk of tumorigenicity (Table 9). Tetrahydroxytricosanoic acid showed low risks of reproductive toxicities and irritation, due to the high number of rotatable bonds (Table 7) in its structure, giving it high reactivity.

## 3. Materials and Methods

### 3.1. Chemicals

A water purification system (Mili-Q Merck Millipore, Chile) was used to obtain ultra-pure water (˂5 µg/L TOC). Methanol (HPLC grade) and formic acid (MS grade) for mass spectrometry analysis were obtained from J.T. Baker (Phillipsburg, NJ, USA). Commercial Folin Ciocalteu reagent, gallic acid, sodium carbonate, 2,4,6-tris(2-pyridyl)-s-triazine, sodium acetate, acetic acid, ferric chloride hexahydrate, hydrochlorid acid, Trolox, Trolox, 2,2′-Azobis(2-amidinopropane) dihydrochloride, phosphate buffer, absolute ethanol, fluorescein solution, acetylcholinesterase (AChE) enzyme, butyrylcholinesterase (BChE) enzyme, acetylcholine, butyrylcholine, Ellman’s reagent (DTNB), galantamine, solution Tris-HCl buffer, sodium chloride, magnesium chloride and HPLC standard with purity higher than 95% (atranol, rhein, evernic acid, barbatic acid, and usnic acid) were obtained from Sigma (Sigma, St. Louis, MO, USA).

### 3.2. Lichen Material

The specimen of the lichens *Lecania brialmontii* (100 g) *Pseudephebe pubescens* (100 g) and *Sphaerophorus globosus* (100 g), were collected by A.T.-B. and M.J.S. on Ardley Island, King George Island, South Shetland Archipelago in February 2021. The specimens were determined by botanist Alfredo Torres-Benítez. Specimen numbers: HL-01032021 (*L. brialmontii*), HL-01022021 (*S. globosus*) and HL-01012021 (*P. pubescens*) were deposited at the Natural Products Laboratory of the Universidad Austral de Chile.

### 3.3. Preparation of the Ethanolic Extracts

About 5 g of each lichen species was macerated with ethanol (three times, 30 mL each time) by ultrasound at room temperature. Each extract was filtered, and the solutions were concentrated under reduced pressure at 38 °C to obtain a gummy extract.

### 3.4. LC Parameters and MS Parameters

The separation and identification of secondary metabolites from the lichens were carried out on a UHPLC-ESI-QTOF-MS system, equipped with UHPLC Ultimate 3000 RS with Chromeleon 6.8 software (Dionex GmbH, Idstein, Germany), and a Bruker maXis ESI-QTOF-MS with the software Data Analysis 4.0 (all Bruker Daltonik GmbH, Bremen, Germany). The chromatographic equipment consisted of a quaternary pump, an autosampler, a thermostated column compartment, and a photodiode array detector. The elution was performed using a binary gradient system with eluent (A) 0.1% formic acid in the water, eluent (B) 0.1% formic acid in acetonitrile, and the gradient: isocratic 1% B (0–2 min), 1–5% B (2–3 min), isocratic 5% B (3–5 min), 5–10% B (5–8 min), 10–30% B (8–30 min), 30–95% B (319–38 min), and 1% B isocratic (39–50 min). The separation was carried out with an acclaim Thermo 5 μm C18 80 Å (150 mm × 4.6 mm) column at a flow rate of 1.0 mL/min. ESI-QTOF-MS experiments in negative ion mode were recorded and the scanning range was between 100 and 1200 *m*/*z*. Electrospray ionization (ESI) conditions included capillary temperature of 200 °C, a capillary voltage of 2.0 kV, dry gas flow of 8 L/min, and a pressure of 2 bars for the nebulizer. The experiments were performed in automatic MS/MS mode. The structural characterization of the bioactive compounds was based on HR full MS, fragmentation patterns, and similarity with literature data. For the analysis, 5 mg of each extract was dissolved in 2 mL of methanol, passed through a polytetrafluoroethylene (PTFE) filter, and 10 µL were injected into the apparatus.

### 3.5. Total Phenolic Content

The total phenolic content (TPC) of *L. brialmontii*, *P. pubescens* and *S. globosus* extracts was measured by the Folin–Ciocalteu method and the AlCl_3_ method, using a Synergy HTX microplate reader (Biotek, Winoosky, VT, USA) [51]. Results were expressed as mg of gallic acid per gram of dried lichen. Each experiment was performed in triplicate and values were expressed with mean and standard deviation.

### 3.6. Antioxidant Activity

#### 3.6.1. Ferric-Reducing Antioxidant Power (FRAP) Assay

The FRAP assay was performed following a method previously described in [52]. The standard used was Trolox, and by interpolation on a calibration curve, the results were expressed as micromoles of Trolox equivalents per gram of dried lichen. All experiments were performed in triplicate, and the data were reported as the mean and its standard deviation.

#### 3.6.2. Oxygen Radical Absorbance Capacity (ORAC) Assay

The ORAC assay was performed following a previously described method in [53]. A calibration curve was prepared with the Trolox standard in reaction with fluorescein. The results were expressed in micromoles of Trolox equivalents per gram of dry lichen. Each experiment was performed in triplicate, and the data were reported as the mean and its standard deviation.

### 3.7. Determination of Cholinesterase Inhibition

The determination of this activity was based on the Ellman method, reported previously in [54]. The galantamine standard was prepared to make the calibration curve. DTNB solution, AChE, and BChE enzyme solution, and the addition of substrates acetyl-thiocholine iodide and butyryl-thiocholine chloride, as appropriate, were used in a microplate. Each experiment was performed in triplicate and values were expressed as µg/mL denoting the IC50 for each sample.

### 3.8. Docking Simulations

Crystallographic enzyme structures of Torpedo Californica acetylcholinesterase (TcAChE; PDBID: code 1DX6 [55]), human butyrylcholinesterase (hBChE; PDBID: code 4BDS [56]) downloaded from the RCSB PDB protein data bank [57,58] were analyzed. Enzyme optimizations were carried out using UCSF Chimera software (v1.16, San Francisco, CA, USA), where water molecules and ligands were removed from crystallographic protein active sites. In the same way, all polar hydrogen atoms were added at pH = 7.4. The appropriate ionization states for basic and acidic amino acid residues were considered. The size of the bounding box was fixed at a cube with sides of length 20 Å. The centroid of the selected residue was chosen based on the putative catalytic site of each enzyme, considering its known catalytic amino acids: Ser200 for acetylcholinesterase (TcAChE) [59,60], Ser198 for butyrylcholinesterase (hBChE) [61,62]. The two-dimensional structures of the ligands were drawn with ChemDraw 8.0 (PerkinElmer Informatics, Waltham, MA, USA) and imported into Avogadro (https://avogadro.cc, accessed on 20 April 2022) to optimize the geometry using the force field function MMFF94 [63,64]. All compounds were saved as mol2 files for further docking studies [65,66,67]. The standard procedure for molecular coupling was performed, using the corresponding rigid crystallographic enzymatic structures and flexible ligands (Barbatic acid, Lecanoric acid, Brialmontin 2, Tetrahydroxytricosanoic acid, Sphaerophorin and Sekikaic acid), whose torsion angles were identified (for 10 independent runs per ligand). Directed coupling was performed using the UCSF Chimera program [68,69], where the catalytic pocket of the reference inhibitor galantamine for acetylcholinesterase and butyrylcholinesterase was used. Polar hydrogens and partial Gasteiger charges were added, and a grid box was created using the Autodock Vina tools at UCSF Chimera. Docking and analysis results were visualized using Discovery Studio Visualizer [70]. Upon completion of coupling, the best conformation for hydrogen bonding or π interactions, including the binding energy of the free ligand (kcal/mol), was analyzed [66,67].

### 3.9. Calculation of ADME Parameters

Osiris Data Warrior toolkits (v 5.5.0) were used to verify the pharmacokinetic properties of the phytochemicals extracted. Molecular descriptors that were calculated are the logarithm of the partition coefficient (cLogP), the number of hydrogen bond donors, number of hydrogen bond acceptors, molecular mass of compounds, topological polar surface area (TPSA), number of spin bonds, and violations Lipinski’s rule of five. Using the TPSA value, the absorption percentage (% ABS) was calculated using the following Equation (1) [50,71]:

% ABS = 109 − (0.345 × TPSA)(1)

### 3.10. Calculation of Risk Toxicity

To calculate the toxicological properties of the phytochemicals, the Osiris Data Warrior computational tool was used. The toxicity risks evaluated in each of the molecules were mutagenicity, tumorigenicity, irritation and reproductive effect [50,72].

### 3.11. Statistical Analysis

The results were expressed as the mean of the data with standard deviation (SD) using GraphPad Prism software, version 5.0. (GraphPad Software, Inc., La Jolla, CA, USA). The determination was performed in triplicate for each sample solution. Statistical significance was set at *p* < 0.05 and were determined by one-way ANOVA.

## 4. Conclusions

This work contributes to the chemical characterization of lichen species present in the Antarctic territory Lecania brialmontii, Pseudephebe pubescens and Sphaerophorus globosus. By UHPLC-ESI-QTOF-MS analysis, 18 compounds were identified in L. brialmontii, 18 compounds in P. pubescens and 14 compounds in S. globosus, corresponding to carbohydrates, acids, anthraquinones, aromatics, lipids, depsides and dibenzofurans. The results showed a relatively low phenolic composition in the species and moderate antioxidant activity; likewise, the cholinesterase inhibitory activity showed good values. Molecular docking analysis with typical compounds of these species showed an important interaction on AChE and BChE enzymes as possible inhibitors. In general, these species represent a promising biological resource for research in the prevention and/or neuroprotection of central nervous system diseases, such as Parkinson’s and Alzheimer’s disease.

## Figures and Tables

**Table 1:** Identification of metabolites in *Lecania brialmontii* by UHPLC-QTOF-MS-MS.

Peak	Tentative Identification	[M−H]^−^	Retention Time (min)	Theoretical Mass (*m*/*z*)	Measured Mass (*m*/*z*)	Accuracy (ppm)	Metabolite Type	MS Ions (ppm)
1	Mannitol	C_6_H_13_O_6_	1.34	181.0712	181.0723	3.9	C	151.0598
2	Citric acid	C_6_H_7_O_7_	3.21	191.0192	191.0184	4.2	Ac	111.0074
3	Swertianin	C_14_H_9_O_6_	10.67	273.0352	273.0349	10.2	Ant	-
4	2,4-Diformyl-3,5-dihydroxytoluene o 2,6-Diformyl-3,5-dihydroxytoluene	C_9_H_7_O_4_	13.28	179.0344	179.0353	4.7	A	107.0488; 135.0437; 151.0386
5	5,7-Dihydroxy-6-methylphtalide	C_9_H_7_O_4_	14.32	179.0344	179.0349	3.9	A	135.0438; 107.0488
6	Atranol *	C_8_H_7_O_3_	19.82	151.0395	151.0401	8.3	A	135.0438; 123.0438; 107.0488
7	Rhein	C_15_H_7_O_6_	20.33	283.02441	283.0221	2.5	Ant	273.011; 242.1745
8	9,10,12,13-Tetrahydroxyheneicosanoic acid	C_21_H_41_O_6_	20.73	389.2903	389.2897	2.5	L	371.2784
9	Tetrahydroxypentacosanedioic acid	C_25_H_47_O_8_	21.2	475.3246	475.3252	2.2	L	-
10	9,10,12,13-Tetrahydroxydocosanoic acid	C_22_H_43_O_6_	21.30	403.3060	403.3028	3.9	L	385.2939; 215.1273
11	Pentahydroxyoxohexacosanoic acid	C_26_H_49_O_8_	21.51	489.3432	489.3403	−5.9	L	403. 3001; 979.6848 (2M-H)
12	Evernic acid *	C_17_H_15_O_7_	21.72	331.0818	331.0809	2.7	d	135.0438; 123.0439; 181.0494; 151.0386; 167.0336; 313.0703
13	Brialmontin 2	C_21_H_25_O_5_	22.31	343.1551	343.1567	2.8	d	123.0432; 313.0721
14	9,10,12,13-Tetrahidroxytricosanoic acid	C_23_H_45_O_6_	22.12	417.3236	417.3189	7.7	L	399.3095
15	9,10,12,13,14,15-Hexahydroxyheptacosenoic	C_27_H_51_O_8_	22.40	503.3584	503.3558	5.0	L	475.3615; 443.3355; 371.0377
16	Methyl-9,10,11,12,13-pentahydroxy-14-oxoheptacosanoate	C_28_H_53_O_8_	22.72	517.3740	517.3685	6.3	L	457.3510; 431.3352
17	Lecanoric acid	C_16_H_13_O_7_	23.81	317.0666	317.0624	−10.92	d	167.034
18	Barbatic acid *	C_19_H_19_O_7_	24.28	359.1131	359.1120	3.1	d	181.0493; 163.0387; 137.0594

* Identified by spiking experiments with an authentic standard compound. C = carbohydrates; Ac = acids; Ant = anthraquinone; A = aromatic; L = lipid; d = depside.

**Table 2:** Identification of metabolites in *Pseudephebe pubescens* by UHPLC-QTOF-MS-MS.

Peak	Tentative Identification	[M−H]^−^	Retention Time (min)	Theoretical Mass (*m*/*z*)	Measured Mass (*m*/*z*)	Accuracy (ppm)	Metabolite Type	MS Ions (ppm)
1	Mannitol	C_6_H_13_O_6_	1.34	181.0712	181.0723	3.9	C	151.0598
2	Citric acid	C_6_H_7_O_7_	3.21	191.0192	191.0184	4.2	Ac	111.0074
3	Azelaic acid	C_9_H_15_O_4_	14.70	187.0775	187.0969	−3.63	L	-
4	Visnagin	C_13_H_9_O_5_	17.30	245.0484	245.0431	−22.2	C	165.0923
5	Khellinol	C_13_H_9_O_5_	19.08	245.0489	245.0431	−23.2	C	165.0914
6	9-Octadecenedioic acid	C_18_H_31_O_4_	19.56	311.2227	311.2228	0.2	L	-
7	Pinellic acid	C_18_H_33_O_5_	20.16	329.2333	329.2345	3.6	L	-
8	Olivetolic acid (2,4-Dihydroxy-6-pentylbenzoate)	C_12_H_15_O_4_	20.40	223.0983	223.0981	0.93	L	165.0923
9	Pentahydroxyoxohexacosanoic acid	C_26_H_49_O_8_	22.17	489.3432	489.3561	−7.8	L	403. 3001; 979.6848 (2M-H)
10	Lecanoric acid	C_16_H_13_O_7_	22.91	317.0666	317.0653	−3.2	d	167.034
11	Tetrahydroxytricosanoic acid	C_23_H_46_O_6_	23.14	417.3221	417.3230	2.0	d	245.0456
12	3,5-Diethoxybenzoic acid	C_11_H_13_O_4_	23.50	209.0822	209.0823	0.47	A	163.0360
13	Sekikaic acid	C_22_H_25_O_8_	24.87	417.1553	417.3171	−4.90	A	247.16944
14	17-Hydroxylinolenic acid	C_18_H_29_O_3_	25.19	293.2122	293.2136	4.9	L	243.19740
15	Porrigenic acid	C_18_H_29_O_4_	25.31	309.2070	309.2091	6.51	L	291.19653
16	Usnic acid *	C_18_H_15_O_7_	26.13	343.0823	343.0822	−0.38	DBF	295.2291; 231.0647; 328.0570
17	18-Hydroxylinoleic acid	C_18_H_31_O_3_	26.87	295.22787	295.22878	2.8	L	-
18	18-Hydroxylinolenic acid	C_18_H_29_O_3_	27.89	293.2122	293.2136	4.7	L	243.19740

* Identified by spiking experiments with an authentic standard compound. C = carbohydrates; Ac = acids; L = lipid; d = depside; A = aromatic; DBF = dibenzofuran.

**Table 3:** Identification of metabolites in *Sphaerophorus globosus* by UHPLC-QTOF-MS-MS.

Peak	Tentative Identification	[M−H]^−^	Retention Time (min)	Theoretical Mass (*m*/*z*)	Measured Mass (*m*/*z*)	Accuracy (ppm)	Metabolite Type	MS Ions (ppm)
1	Mannitol	C_6_H_13_O_6_	1.34	181.0712	181.0705	3.9	C	151.0598
2	Vanillic acid	C_8_H_8_O_4_	13.25	167.0749	167.0754	3.0	A	123.0448
3	Vanilloloside	C_14_H_19_O_8_	14.23	315.1085	315.1059	−8.32	A	162.9945
4	Methyl orsellinate	C_9_H_9_O_4_	20.23	181.0501	181.0507	0.5	A	151.0387; 123,0439; 135.0438
5	2,6-Diformyl-3,5-dihydroxytoluene	C_9_H_7_O_4_	20.81	179.0344	179.0338	3.4	A	151.0386; 107.0488; 135.0437
6	Protolichesterinic acid	C_19_H_31_O_4_	21.80	323.2222	323.2213	2.8	Ac	279.2315; 267.2314
7	4′-*O*-methyl norhomosekikaic acid	C_23_H_27_O_8_	22.24	431.1657	431.1681	6.6	d	417.15290; 401.08231; 267.1228
8	Sphaerophorin	C_23_H_27_O_7_	22.80	415.1757	415.1744	3.1	d	233.1166; 207.1376; 251.1275
9	6-Heptylresorcylic acid	C_14_H_19_O_4_	23.56	251.1288	251.1320	12.6	A	207.1403
10	Lecanoric acid	C_16_H_13_O_7_	24.72	317.0666	317.0668	0.45	d	251.13175; 213.7944
11	Sekikaic acid	C_22_H_25_O_8_	25.09	417.1554	417.15290	−6.2	d	267.1228; 251.1289; 285.09033
12	Usnic acid *	C_18_H_15_O_7_	26.13	343.0823	343.0822	−0.38	DBF	295.2291; 231.0647; 328.0570
13	Acetoxyisovalerylalkannin	C_23_H_25_O_8_	27.99	429.1514	429.1545	−2.18	A	167.0360; 251.1298
14	2′-*O*-methyldivaricatic acid	C_22_H_25_O_7_	29.09	401.1605	401.1606	0.1	A	251.1321; 167.0358

* Identified by spiking experiments with an authentic standard compound. C = carbohydrates; A = aromatic; Ac = acids; d = depside; DBF = dibenzofuran.

**Table 4:** Total phenolic content (TPC) and antioxidant activity (FRAP; ORAC) of *Lecania brialmontii*, *Pseudephebe pubescens* and *Sphaerophorus globosus*.

Assay	TPC (mg AG/g)	FRAP (µmol Trolox/g)	ORAC (µmol Trolox/g)
*L. brialmontii*	0.279 ± 0.005 *	45.089 ± 0.002	219.334 ± 0.75 *
*P. pubescens*	0.579 ± 0.01 *	46.422 ± 0.004	146.359 ± 0.56 *
*S. globosus*	2.821 ± 0.08 *	16.662 ± 0.004 *	254.118 ± 0.82 *

The values represent the means ± SD of three replicates (n = 3). Values marked with * are statistically different (*p* < 0.05).

**Table 5:** Enzyme inhibitory activity of *Lecania brialmontii*, *Pseudephebe pubescens* and *Sphaerophorus globosus*.

Assay	AChE IC_50_ (µg/mL)	BChE IC_50_ (µg/mL)
*L. brialmontii*	3.949 ± 0.04 *	4.476 ± 0.06 *
*P. pubescens*	2.805 ± 0.07 *	8.828 ± 0.08 *
*S. globosus*	10.422 ± 0.08 *	6.785 ± 0.04 *
Galanthamine *	0.26 ± 0.02 *	3.82 ± 0.02 *

The values represent the means ± SD of three replicates (n = 3). Values marked with * are statistically different (*p* < 0.05). AChE, acetylcholinesterase and BChE, butyrylcholinesterase. * Positive control.

**Table 6:** Binding energies resulting from molecular docking experiments of the selected compounds in the extracts of *Lecania brialmontii*, *Pseudephebe pubescens* and *Sphaerophorus globosus*, together with the standard inhibitor galantamine on acetylcholinesterase (TcAChE) and butyrylcholinesterase, (hBChE).

Compound.	Binding Energy (Kcal/mol) Acetylcholinesterase (TcAChE)	Binding Energy (Kcal/mol) Butyrylcholinestarase (hBChE)
Barbatic Acid	−10.30	−8.80
Lecanoric Acid	−9.90	−9.40
Brialmontin 2	−9.80	−9.10
Tetrahydroxytricosanoic Acid	−7.90	−6.60
Sphaerophorin	−9.50	−8.70
Sekikaic Acid	−9.30	−8.30
Galanthamine	−10.80	−8.80

**Table 7:** Pharmacokinetic properties of compounds in the extracts of *Lecania brialmontii*, *Pseudephebe pubescens* and *Sphaerophorus globosus* in comparison with the standard inhibitor galanthamine on acetylcholinesterase (TcAChE) and butyrylcholinesterase (hBChE) obtained from Osiris Data Warrior software.

Compound	%ABS ^a^	TPSA (Å^2^) ^b^	MW ^c^	cLogP ^d^	HBD ^e^	HBA ^f^	*n*-ROTB ^g^	Violation of Lipinski’s Rule
Rule	-	-	<500	≤5	≤5	≤10	≤10	≤1
Barbatic Acid	69.91	113.29	360.36	3.19	3	7	5	0
Lecanoric Acid	66.12	124.29	318.28	2.23	4	7	4	0
Brialmontin 2	86.58	64.99	358.43	4.67	1	5	5	0
Tetrahydroxytricosanoic Acid	68.21	118.22	418.61	6.95	5	6	21	2
Sphaerophorin	69.91	113.29	416.47	5.19	3	7	11	1
Sekikaic Acid	66.73	122.52	418.44	4.17	3	8	10	0
Galanthamine *	94.53	41.93	287.35	1.19	1	4	3	0

Note: ^a^ Percentage of absorption (%ABS); ^b^ topological polar surface area (TPSA); ^c^ molecular weight (MW); ^d^ logarithm of partition coefficient between *n*-octanol and water (cLogP); ^e^ number of hydrogen bond donors (HBD); ^f^ number of hydrogen bond acceptors (HBA); ^g^ number of rotable bonds (*n*-ROTB). * Compound recognized as a standard inhibitor.

**Table 8:** Calculation of toxicity risks of compounds in the extracts of *Lecania brialmontii, Pseudephebe pubescens* and *Sphaerophorus globosus* in comparison with the standard inhibitor galanthamine on acetylcholinesterase (TcAChE) and butyrylcholinesterase (hBChE) obtained from Osiris Data Warrior software.

Compound	Mutagenic	Tumorigenic	Irritant	Reproductive Effect
Galanthamine	None	None	None	None
Barbatic Acid	None	None	None	None
Lecanoric Acid	None	None	None	None
Brialmontin 2	None	High	High	None
Tetrahydroxytricosanoic acid	None	None	Low	Low
Sphaerophorin	None	None	None	None
Sekikaic Acid	None	None	None	None

**Table 9:** Structural fragments responsible for toxicity in the extracted compounds from *Lecania brialmontii*.

Compound	Fraction of Molecule	Risk of Toxicity
Brialmontin 2	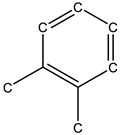	High-risk fragment indicating Tumorigenicity
	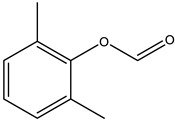	High-risk fragment indicating Irritating

## Data Availability

The data presented in this study are available on request from the correspondings authors.

## Sample Availability

Not available.
